# Establishment and Application of Mismatch Amplification Mutation Assay-PCR for Rapid Detection and Differentiation of Duck Hepatitis A Virus-1 Attenuated Vaccine and Wild Strains

**DOI:** 10.3390/ani14182733

**Published:** 2024-09-21

**Authors:** Cheng-Dong Yu, Yu-Ri Choi, Jong-Yeol Park, Sang-Won Kim, Se-Yeoun Cha, Hyung-Kwan Jang, Min Kang, Bai Wei

**Affiliations:** 1 Department of Avian Diseases, College of Veterinary Medicine and Center for Avian Disease, Jeonbuk National University, Iksan 54596, Republic of Korea; yuchengdong@naver.com (C.-D.Y.); 9cinderella7@naver.com (Y.-R.C.); jyp410@naver.com (J.-Y.P.); sk970221@gmail.com (S.-W.K.); seyeouncha@jbnu.ac.kr (S.-Y.C.); hkjang@jbnu.ac.kr (H.-K.J.); 2 Bio Disease Control (BIOD) Co., Ltd., Iksan 54596, Republic of Korea

**Keywords:** DHAV-1, MAMA-PCR, differentiation, wild-type, attenuated vaccine

## Abstract

**Simple Summary:**

Duck hepatitis A virus (DHAV) is an infectious pathogen that causes fatal viral hepatitis in ducklings. Duck hepatitis A virus-1 (DHAV-1) is the most virulent and widely distributed serotype, inflicting severe damage on the global duck farming industry. The main approach to preventing DHAV-1 involves using attenuated live vaccines. However, these vaccine strains may persist in ducks and pose a risk of reverting to virulence. Therefore, distinguishing between infected and vaccinated animals is vital for clinical diagnosis and vaccine safety monitoring. Currently, only time-consuming and costly sequencing methods serve this purpose. In this study, a Mismatch Amplification Mutation Assay (MAMA)-PCR method was developed based on single nucleotide polymorphism (SNPs) in the VP1 gene. The MAMA-PCR method offers a highly specific and sensitive clinical tool for rapidly distinguishing DHAV-1 wild-type from vaccine strains, significantly contributing to accurate clinical diagnosis and epidemiological understanding.

**Abstract:**

Duck hepatitis A virus type 1 (DHAV-1) is the main pathogen causing viral hepatitis in ducks, marked by high contagion and acute mortality. Live attenuated DHAV-1 vaccines are widely used to control the disease. This study aims to develop a mismatch amplification mutation assay (MAMA)-PCR for the rapid detection and differentiation of Korean DHAV-1 wild-type strains from vaccine strains. A MAMA primer was designed to target a single nucleotide polymorphism (SNPs) at position 2276 within the VP1 gene, allowing differentiation in a single PCR reaction. The MAMA-PCR accurately identified both strains, with detection limits of 10^0.5^ ELD_50_/mL and 10^2.3^ ELD_50_/mL, respectively. The MAMA-PCR demonstrated specificity, showing no cross-reactivity with 12 other viral and bacterial pathogens. The MAMA-PCR was applied to 89 farms, yielding results consistent with nested-PCR and sequence determination, identifying four positive farms for DHAV-1 vaccine strains. In conclusion, this study is the first to employ the MAMA-PCR method to distinguish between DHAV-1 wild-type and vaccine strains. The developed method is rapid, simple, specific, and sensitive, thereby serving as an effective tool for clinical diagnostics in identifying and differentiating between Korean DHAV-1 wild-type and vaccine strains.

## 1. Introduction

Duck hepatitis is an acute and highly contagious disease that primarily affects ducklings, particularly those under 6 weeks of age, often resulting in mortality rates as high as 95–100% [1,2]. The causative agent, duck hepatitis A virus (DHAV), is classified under the *Avihepatovirus* genus within the *Picornaviridae* family, according to the latest classification by the International Committee on Taxonomy of Viruses (Virus Taxonomy: 2020 Release). DHAV is classified into three distinct serotypes: DHAV-1, DHAV-2, and DHAV-3 [2]. DHAV-1—the most prevalent and virulent serotype—induces acute symptoms in ducklings, such as lethargy, ataxia, and opisthotonos [1]. The DHAV-1 genome comprises a single, long, open reading frame (ORF) that encodes a polyprotein. This polyprotein is predicted to consist of three structural proteins (VP0, VP1, and VP3) and nine non-structural proteins (2A1, 2A2, 2A3, 2B, 2C, 3A, 3B, 3C, and 3D) [3,4]. The VP1 protein is the primary antigen that induces neutralizing antibody production and plays a crucial role in receptor binding and virulence among these proteins [5,6,7,8].

Vaccination is the primary strategy for preventing DHAV-1 infection, with live attenuated vaccines preferred for their rapid onset of immunity [8]. However, vaccine strains of DHAV-1 can persist in vaccinated individuals [9]. Therefore, differentiating infected from vaccinated animals (DIVA) is crucial for preventing economic losses resulting from mistakenly culling vaccinated ducks. Currently, molecular biological methods for DIVA of DHAV-1 are limited. Sequencing is the only tool available to distinguish vaccine strains from wild-type strains, however, it is time-consuming and costly [10]. Rapid and accurate detection and DIVA of DHAV-1 are essential for effective prevention and control.

Live attenuated DHAV vaccines are produced by attenuating virulent strains through continuous passage in chicken or duck embryos [8]. The attenuation of DHAV-1 during passage is associated with specific site changes within the VP1 protein [11]. The sequence analysis of five chicken embryo-attenuated DHAV-1 strains and sixty-eight virulent strains has revealed multiple conserved single nucleotide polymorphisms (SNPs) distinguishing vaccine strains from virulent ones [12]. SNPs are biologically significant point mutations widely utilized in clinical applications [13]. Mismatch amplification mutation assays-PCR (MAMA-PCR) target SNP-specific sites at the 3′ end of primers, creating mismatches between primers and templates for differentiation [14,15]. MAMA-PCR offers advantages owing to its experimental conditions, design complexity, time efficiency, and cost-effectiveness, making it suitable for most basic laboratories [13,16].

DHAV-1 has been detected in several countries, including China, South Korea, Vietnam, the United States, the United Kingdom, Poland, and Egypt [17]. The first report of DHAV-1 in South Korea dates back to 1985, followed by the development and clinical introduction of the HSB-P100-attenuated vaccine by 2000 [18,19]. Despite extensive vaccination efforts, DHAV-1 continues to persist in the duck population, causing significant economic losses in the duck farming industry in South Korea [20]. Owing to the rapid onset and high mortality of DHAV-1, this study aims to develop a MAMA-PCR method for the rapid detection of DHAV-1 strains and differential diagnosis of the HSB-P100 vaccine strain from non-HSB-P100 strains.

## 2. Materials and Methods

### 2.1. Virus Strains

The commercially available vaccine strain HSB-P100 (GenBank accession number, PP592357), currently utilized in Korea, and field isolate ATCC: VR-1313 (GenBank accession number, DQ219396), were used to develop the MAMA-PCR method. Previously reported field strains DHAV-HS (GenBank accession number, DQ812094) and DHAV-HSS (GenBank accession number, DQ812092), isolated in Korea, were used to assess the reliability of the established method. The viruses were propagated in the allantoic cavity of 8-day-old specific pathogen-free (SPF) chicken embryos (Sunrise Farms, Inc., Stuarts Draft, VA, USA) at 37 °C for 5 days [9]. After two passages, the harvested viruses were stored at −70 °C until needed for further use. The 50% embryo lethal dose (ELD_50_) was calculated using this method [21].

### 2.2. Sequence Analysis and Primer Design

**Figure 1:**
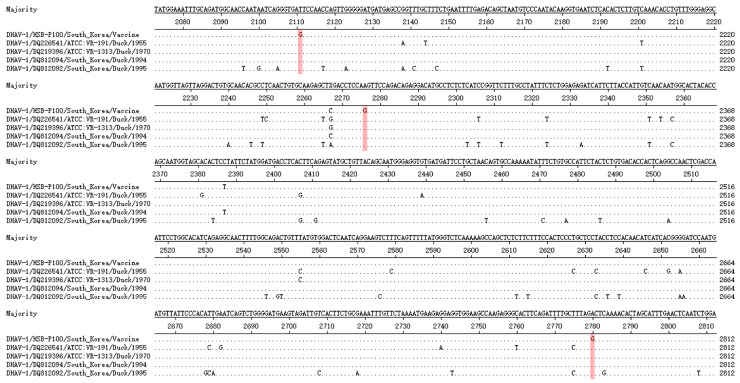
Multiple sequence alignment between the DHAV-1 vaccine and wild-type strains. The Clustal W alignment method in the MegAlign module of DNASTAR software was used to align the sequences of vaccine strain HSB-P100 with four wild-type strains from ATCC, Korea. Three specific SNPs were found in the vaccine strain at nucleotide positions 2111th, 2276th, and 2780th. The red region indicates these positions in each sequence. “.” indicates nucleotides identical to those in HSB-P100.

The complete genome of four DHAV-1 field isolates (ATCC and South Korea) was obtained from the NCBI GenBank database. Multiple sequence alignments were conducted using the MegAlign module of DNASTAR software (https://www.dnastar.com/). Primers DHAV-1-C1 and DHAV-1-C2 for DHAV-1 detection were designed from the highly conserved region of the VP1 gene. Nucleotide polymorphisms between the Korean vaccine strain HSB-P100 and other DHAV-1 field isolates were analyzed (Figure 1). The MAMA-F1 and MAMA-R1 primers were designed to incorporate the vaccine strain-specific SNPs’ site as their 3′ ends, enabling differentiation between wild-type and the vaccine strain. Table 1 shows the primer sequences, and all primers were synthesized by Bioneer Corporation (Daejeon, Republic of Korea).

### 2.3. RNA Extraction and Reverse Transcription

The Viral Gene-spin™ Viral DNA/RNA Extraction Kit (iNtRON, Daejeon, Korea) was used to extract the RNA needed for the experiment following the instruction of the manufacturer. In the reverse transcription (RT) steps, 1 μL of random primer was added to every 10 μL of extracted RNA, heated at 70 °C for 5 min, followed by cooling in an ice bath for 5 min. Subsequently, a mixture was prepared to contain 4 μL of GoScript™ 5X RT reaction buffer (Promega, Madison, WI, USA), 1 µL of 10 mM dNTP, 1.5 μL of 25 mM MgCl_2_ (Promega, Charbonnie, France), 0.5 μL of 40 units Recombinant RNasin^®^ Ribonuclease Inhibitor (Promega, Madison, WI, USA), 1 μL of GoScript™ reverse transcriptase (RTase, Promega, Madison, WI, USA), and 1 μL of diethylpyrocarbonate-treated water (DEPC water, Biosesang, Yongin, Korea). The RT reaction, totaling 20 μL, was incubated at 25 °C for 5 min, followed by 42 °C for 1 h. Finally, to deactivate the RTase, it underwent a 70 °C incubation for 15 min.

### 2.4. Establishment of MAMA-PCR Method

To distinguish between the wild-type and vaccine strains of DHAV-1, MAMA-PCR was conducted using the C1000 Touch™ Thermal Cycler (Bio-Rad, Hercules, CA, USA). PCR reactions were conducted using 10X e-Taq Reaction Buffer (Solgent, Daejeon, Korea), 2 mM dNTP Mix (Solgent, Daejeon, Korea), and Solg™ e-Taq DNA Polymerase (5 U/μL, Solgent, Daejeon, Korea). Initially, the discrimination primers MAMA-F1 and MAMA-R1 were evaluated for their effectiveness. Subsequently, optimal concentrations of MAMA-F1, DH1-VP1-C1, and DH1-VP1-C2 primers were established, along with optimizing the ideal Mg^2+^ concentration. Finally, the number of amplification cycles, annealing temperature and time, and extension time were systematically adjusted to optimize the reaction conditions in the PCR program.

### 2.5. Specificity and Sensitivity of MAMA-PCR

The primers were searched using NCBI BLAST (https://blast.ncbi.nlm.nih.gov/Blast.cgi, accessed on 26 February 2024) to confirm their specificity. Twelve common pathogens in ducks were selected to assess the specificity of the developed MAMA-PCR. These included six viral pathogens: duck hepatitis A virus-3 (DHAV-3), duck enteritis virus (DEV), duck parvovirus (DPV), duck circovirus (DuCV), avian influenza virus (AIV), duck Tembusu virus (DTMUV), as well as six bacterial and mycoplasmal pathogens: *Riemerella anatipestifer* (RA), *Pasteurella multocida* (PM), Avian Pathogenic *Escherichia coli* (APEC), *Clostridium perfringens* (CP), *Mycoplasma gallisepticum* (MG), and *Mycoplasma synovialis* (MS).

To assess the sensitivity of the developed method, samples of the wild-type and vaccine strain were subjected to 10-fold serial dilution with deionized water, after which RNA extraction was performed on the diluted samples. The detection range for wild-type strain samples was from 10^4.3^ to 10^0.3^ ELD_50_/mL, while for vaccine strain samples the detection ranged from 10^3.5^–10^−1.5^ ELD_50_/mL.

### 2.6. Detection of Clinical Samples

Between 2013 and 2022, selected samples from 89 farms located in the main duck-raising regions of South Korea (Jeollabuk-do, Jeollanam-do, Chungcheongbuk-do, Chungcheongnam-do, and Gyeonggi-do) were submitted to the Center for Avian Disease for DHAV diagnosis. Each farm provided approximately 5–20 dead or clinically symptomatic live ducklings for DHAV diagnosis. These ducklings exhibited typical clinical signs and gross lesions indicative of DHAV infections, including lethargy, spasmodic movements, and opisthotonos, as well as petechial and ecchymotic hemorrhages in their enlarged livers. Liver samples were collected and stored at −70 °C until further analysis. All animal experiments were conducted in compliance with the requirements of the National Association of Laboratory Animal Care and Ethics Committee of Jeonbuk National University (Approval No: JBNU 2020-0162).

Homogenized suspensions at 20% were prepared using PBS supplemented with 1% antibiotic-antimycotic (Invitrogen, Carlsbad, CA, USA). These suspensions were then centrifuged at 13,000 rpm for 10 min at 4 °C. Viral RNA was extracted from the resulting supernatant and subjected to reverse transcription. A standard nested PCR detection method for DHAV-1 was used in Korea, and the developed MAMA-PCR was employed simultaneously to identify DHAV-1 in clinical samples [20]. Furthermore, PCR-positive samples underwent sequencing to differentiate between wild-type and vaccine strains.

## 3. Results

### 3.1. Establishment of the MAMA-PCR

The analysis of the multiple sequence alignment results revealed that the vaccine strain HSB-P100 exhibited specific nucleotide mutations at positions 2111th, 2276th, and 2780th within the VP1 gene compared to those of wild-type strains. The nucleotide positions 2111th and 2780th are situated at both ends of the VP1 gene, respectively. Designing primers for these sites could affect the length and efficiency of amplified fragments when used alongside detection primers. Therefore, nucleotide position 2276th was chosen as the SNP site for the MAMA primers. The MAMA-F1 primer was designed with the 2276th nucleotide polymorphic site (G) at the 3′ end, with an additional mismatch site (C→A) introduced at the antepenultimate position. Similarly, the MAMA-R1 primer also utilizes the 2276th nucleotide polymorphic site as the 3′ end, incorporating an additional mismatch site (A→T) at the antepenultimate position. In comparison, the band produced by the MAMA-F1 primer showed greater intensity in differentiating between wild-type and vaccine strains, leading to its selection for method development.

The PCR reaction system, with a total volume of 25 μL, included 2.5 μL of 10× e-Taq Reaction Buffer, 1.5 μL of 2 mM dNTP Mix, and 0.25 μL of Solg™ e-Taq DNA Polymerase. For detection, 1 μL of the forward primer DH1-VP1-C1 (10 pmol) and 1.5 μL of the reverse primer DH1-VP1-C2 (10 pmol) were used, along with 3 μL of the MAMA-F1 primer (10 pmol) designed for distinguishing between the wild-type and vaccine strains. The remaining 13.25 μL consisted of ddH_2_O. The PCR reaction conditions were as follows: initial denaturation at 94 °C for 5 min, followed by 35 cycles of denaturation at 94 °C for 1 min, annealing at 63 °C for 1 min, and extension at 72 °C for 45 s. The final extension step was conducted at 72 °C for 15 min. The amplified DNA was analyzed via electrophoresis on a 1.5% agarose gel.

**Figure 2:**
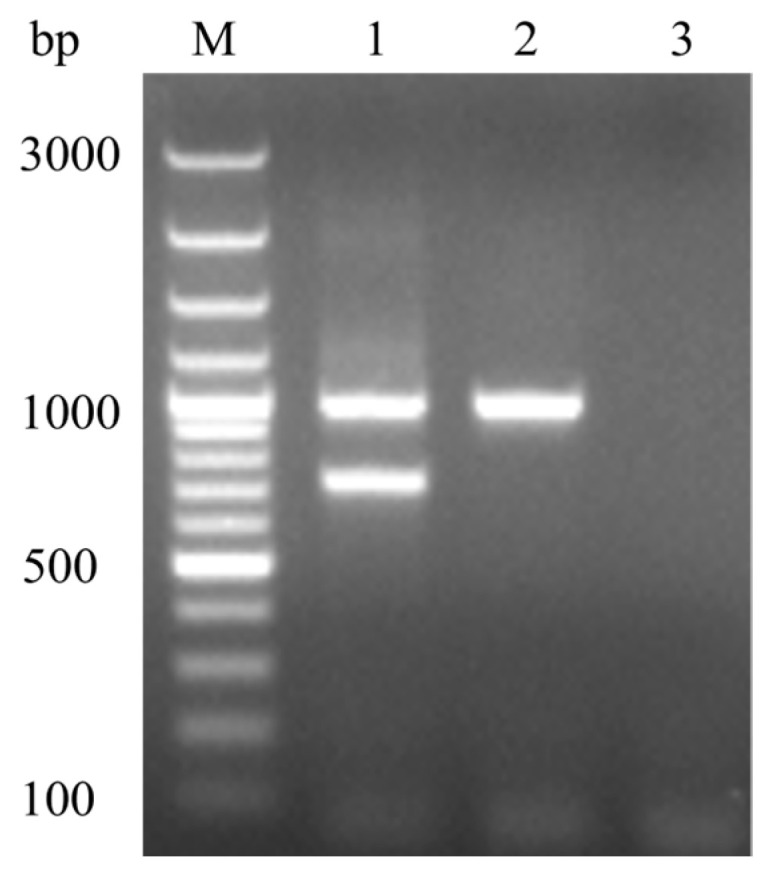
MAMA-PCR for identifying and distinguishing the vaccine strain from the wild-type strain. M: 100 bp DNA marker; Lane 1: vaccine strain; Lane 2: wild-type strain; Lane 3: negative control. The vaccine strain exhibits two distinct bands at 986 bp and 722 bp, while the wild-type strain shows a single band at 986 bp.

PCR analysis revealed two distinct bands at 986 bp and 722 bp for the DHAV-1 vaccine strain. Conversely, the wild-type strain exhibited a single band at 986 bp. No apparent non-specific bands were observed, and the negative control showed no bands (Figure 2).

### 3.2. Evaluation of MAMA-PCR Specificity and Sensitivity

**Figure 3:**
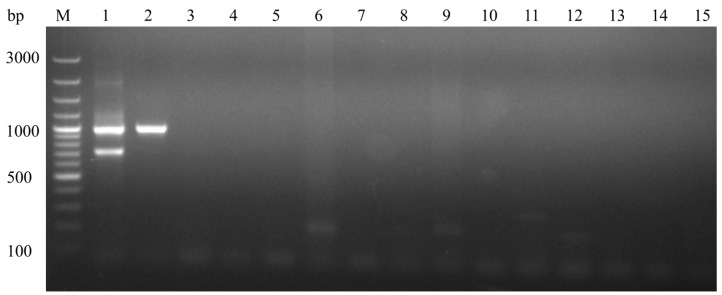
Specificity of the MAMA-PCR. M: 100 bp DNA marker; Lane 1: vaccine strain; Lane 2: wild-type strain; Lane 3: DHAV-3; Lane 4: DEV; Lane 5: DPV; Lane 6: DuCV; Lane 7: AIV; Lane 8: DTMUV; Lane 9: RA; Lane 10: PM; Lane 11: APEC; Lane 12: CP; Lane 13: MG; Lane 14: MS; Lane 15: negative control.

The BLAST search results showed that the detection primers used exhibited 100% homology with the DHAV-1 sequence. However, the distinguishing primer MAMA-F1 did not exhibit 100% homology with any sequence. Except for the additional introduced mismatch site, the primer showed 100% homology only with the HSB-P100 sequence, demonstrating its high specificity. During validation of the PCR method using DNA or RNA extracted from DHAV-3, DEV, DPV, AIV, DTMUV, RA, PM, APEC, CP, MG, and MS, no target fragments were observed, confirming the specificity of the method. The assay showed no cross-reactivity with the 12 common viral, bacterial, and mycoplasmal diseases in ducks (Figure 3). These findings indicate that the developed MAMA-PCR assay, designed specifically for DHAV-1, exhibits excellent specificity.

**Figure 4:**
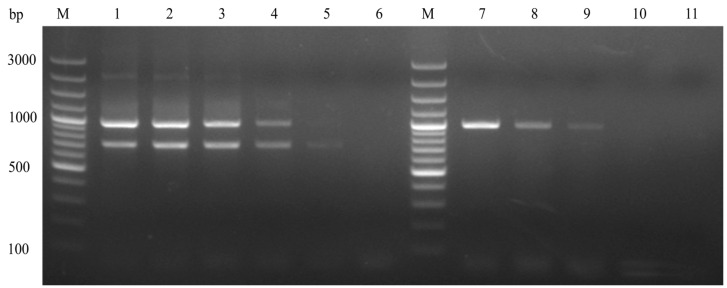
Sensitivity test of MAMA-PCR. M: 100 bp DNA marker; Lanes 1~6: vaccine strain 10^3.5^~10^−1.5^ ELD_50_/mL; Lanes 7~10: wild-type strain 10^4.3^~10^1.3^ ELD_50_/mL; Lane 11: negative control.

Samples of DHAV-1 wild-type and vaccine strains, each with known concentrations, underwent ten-fold serial dilutions. Sensitivity tests were conducted using RNA extracted from these samples at various dilutions. The results showed that the MAMA-PCR method achieved detection limits of 10^2.3^ ELD_50_/mL and 10^0.5^ ELD_50_/mL for wild-type and vaccine strains, respectively (Figure 4).

### 3.3. Detection in Clinical Samples

The detection results obtained from clinical samples indicated complete consistency between the developed MAMA-PCR- and DHAV-1-nested PCR results. Among the samples collected from 89 farms, samples from four farms tested positive for DHAV-1 (Table 2). In MAMA-PCR detection, all positive samples exhibited distinct double bands at 986 bp and 722 bp. Based on the PCR results, all positive samples were confirmed to be infected with the DHAV-1 vaccine strain. Subsequently, the VP1 gene of PCR-positive samples underwent sequencing and analysis. The sequencing analysis revealed that the four samples exhibited identical mutation sites in the VP1 gene, similar to those observed in the HSB-P100 vaccine strain (Supplementary Table S1). This further confirmed infection with the DHAV-1 vaccine strain.

## 4. Discussion

Strict biosecurity measures and effective vaccination programs are fundamental for preventing and controlling DHAV, as with all infectious diseases in animals [8]. Owing to the rapid onset and high mortality of DHAV-1, the conventional sequencing method is hindered by its time-consuming and expensive nature, rendering it unsuitable for widespread clinical application. Therefore, this study aimed to address the limitations of conventional sequencing methods by developing a simple MAMA-PCR method for rapidly distinguishing DHAV-1 vaccine strains from wild-type strains.

The DHAV-1 strains have been shown to exhibit regional specificity, and there is currently no evidence of transmission between Korea and other countries, further indicating the geographic restriction of DHAV-1 spread within Korea [17]. Therefore, vaccines are typically developed based on strains prevalent in specific regions, and in Korea only the HSB-P100 strain is currently in use [8]. In this study, we developed a MAMA-PCR method specifically designed to distinguish between vaccine and wild-type strains of DHAV-1 in Korea, targeting specific nucleotide variations within the VP1 gene, a key determinant of DHAV-1 virulence [11,22]. The identified variations at positions 2111 (T→G), 2276 (A→G), and 2780 (A→G) are crucial for distinguishing the Korean vaccine strain HSB-P100 from wild-type strains. The specific mutation (A2276G) in the VP1 gene of the Korean DHAV-1 vaccine strain is located at the 3′ end of the MAMA-PCR primer. Mismatches at this position destabilize primer-template annealing, reducing Taq polymerase extension efficiency and consequently lowering PCR efficiency [23]. The effects on amplification efficiency vary depending on the nature of the base mismatch at the 3′ end of primers. Compared to amplification with a perfect match between the 3′ end of the primer and template, the amplification efficiency per cycle ranges from 50% to 16% (during the first 10 cycles) [24]. In this study, an additional mismatched position (C→A) was introduced at the antepenultimate position of the 3′ end of the primer, enhancing the selectivity of SNP-allelic discrimination by further hindering the extension of the mismatched primer [25]. In conclusion, during the amplification of the vaccine strain, the SNP site at the 3′ end of the primer can perfectly match the vaccine strain template, resulting in double bands at 986 bp and 722 bp. In contrast, a single band at 986 bp was generated for the wild-type strain.

The developed MAMA-PCR is a rapid, sensitive, and specific method for differentially detecting vaccine strains from wild-type strains. This study demonstrated high sensitivity, detecting the wild-type strain at a limit of 10^2.3^ ELD_50_/mL, consistent with previous DHAV detection methods [26]. Furthermore, our novel MAMA-PCR detected the vaccine strain with a low limit of 10^0.5^ ELD_50_/mL. The increased sensitivity allows for effective detection and differentiation of low levels of vaccine strain in the field. The attenuated vaccine strain did not exhibit the same replication ability as the wild-type strain in vivo [9,27]. In addition, the developed MAMA-PCR also exhibited high specificity, avoiding cross-reactions with common duck pathogens. This capability meets practical application needs for the differential detection of vaccine strains from wild-type strains. DHAV-1 frequently co-infects with other viral and bacterial pathogens, especially those highly concurrent with DHAV-3, DuCV, RA, and APEC [28,29,30,31]. The developed MAMA-PCR method did not amplify specific bands for any of these 12 viral and bacterial pathogens, meeting clinical requirements.

Here, the MAMA-PCR detection results were fully consistent with the findings of the sequencing analysis. This indicates that MAMA-PCR facilitates rapid and accurate diagnosis. It also aids in monitoring the influence of vaccination programs on disease spread [14,32]. All four positive DHAV-1 samples were identified as DHAV-1 vaccine strains. These findings align with the absence of reported DHAV-1 outbreaks in Korea since its last detection in 2013 [20]. In addition, the vaccine strain identified in this study aligns with that of previous studies in that the live attenuated vaccine strain can replicate in the internal organs of ducks and be shed into the environment, potentially infecting non-immunized ducklings [9,33]. Our results also align with previous findings, indicating that DHVA-1 vaccine strains exhibit stability in the farm environment and high transmissibility. This is demonstrated by the identification of DHAV-1 vaccine strains in duck farms (D15-MR-038 & D18-ETC-001) without a history of vaccination (Supplementary Table S1). Furthermore, the live attenuated vaccine strain persists in the duck farm environment, and it can be transmitted to the next batch of non-immunized ducklings (D18-ETC-001). These results could also be supported by the known stability of DHAV in wet environments, as the DHAV-1 virus can survive in moist feces for over 37 days [34].

All samples identified as DHAV-1 vaccine strains showed consistent liver and kidney lesions in deceased ducks. Among the four positive samples, only one showed co-infection with *Salmonella*, while the other three did not show co-infection with common duck pathogens such as DEV, DuCV, RA, APEC, and *Salmonella* (Supplementary Table S2). The exact cause of the hepatitis-like symptoms remains undetermined. Attenuated vaccines typically do not result in clinical signs or mortality in ducklings [9]. However, co-infection with viral, bacterial, and/or immunosuppressive agents can lead to severe disease, resulting in higher mortality rates [35,36,37]. In addition to DHAV-1, both DHAV-2 and DHAV-3 have the potential to cause similar clinical symptoms. DHAV-3 infections are relatively common in Korean duck farms, and co-infections with DHAV-1 have been reported [20]. However, DHAV-2 has not yet been detected in Korea, and its presence has been geographically limited to regions such as Taiwan and India [8,38]. As such, while our study focused on DHAV-1, future diagnostic methods should also include DHAV-2 and DHAV-3 to ensure comprehensive monitoring and control of duck viral hepatitis in Korea. To further investigate the potential causes of the clinical signs observed in the remaining samples, additional diagnostic tests were conducted on the samples from 85 farms that exhibited DHAV-1-like clinical signs. Common pathogens, including AI, DEV, DPV, DTMUV, DuCV, RA, APEC, and MG were tested. Among these, DuCV, RA, APEC, and MG were detected, with RA and APEC showing the highest infection rates. Moreover, infections with common bacterial pathogens can compromise the intestinal barrier function in ducks, exacerbating the pathogenic effects of a single infection [39]. Further studies should also investigate other duck pathogens resulting in hepatitis-like symptoms not yet identified in Korea [40]. Another potential consideration is the safety of live attenuated vaccines. Current live vaccines undergo extensive attenuation through multiple passages in chicken or duck eggs, ensuring safety. No virulence reversion has been observed after several rounds of back passage in ducks [41]. However, Woolcock PR et al. [42] indicated that the virulence of three attenuated DHAV-1 vaccine strains (H55, Q50, and C-MLV85) is enhanced through serial passage in susceptible ducklings. In another study, two attenuated DHAV strains passaged approximately 90 times in chicken embryos and were found to revert to virulence through serial passage in ducklings [43]. Therefore, the risk of virulence reversion in live attenuated vaccines cannot be completely excluded, especially under field conditions. Continuous monitoring of the safety of live DHAV vaccines in duck flocks is recommended.

## 5. Conclusions

In summary, the MAMA-PCR method developed in this study offers a highly specific and sensitive tool for distinguishing DHAV-1 wild-type and vaccine strains. By addressing the limitations of conventional methods and introducing a simplified approach, our study findings significantly contribute to facilitating accurate clinical diagnosis and epidemiological understanding. Furthermore, the developed method also reduces the economic risks associated with delayed detection or misdiagnosis in clinical settings, underscoring its importance in preventing and controlling DHAV-1 in clinical practice.

## Figures and Tables

**Table 1:** Primers used for MAMA-PCR.

Group	Primer	Sequences (5′-3′)	Target Gene	Wild-Type Strain Size (bp)	Vaccine Strains Size (bp)
1	MAMA primer-F1	CTGTGCAAGAGCTCGACCTCAAG	VP1	986	986 and 722
	DH1-VP1-C1	CTGTGAATTCATCAGCCCCAT			
	DH1-VP1-C2	ACGTGGTGACAGTTTTGATTC			
2	MAMA primer-R1	GCATGTCCTCTGTCTGGATCC			986 and 307
	DH1-VP1-C1	CTGTGAATTCATCAGCCCCAT			
	DH1-VP1-C2	ACGTGGTGACAGTTTTGATTC			

**Table 2:** Clinical sample analysis results using the MAMA-PCR and VP1 sequencing.

VP1 Sequence (No.)		MAMA-PCR (No.)			
		Vaccine	Wild	Negative	Total
Vaccine	4	4	0	0	4
Wild	0	0	0	0	0
Negative	85	0	0	85	85
Total	89	4	0	85	89

## Data Availability

The data presented in this study are available from the corresponding author on reasonable request.

## Supplementary Materials

The following supporting information can be downloaded at: https://www.mdpi.com/article/10.3390/ani14182733/s1, Supplementary Table S1. Comparison of VP1 gene nucleotide-specific mutation sites among ATCC strain, Korean wild strains, Korean vaccine strain, and clinical strains. Supplementary Table S2. Summary of characteristics of four clinically positive samples.
