# Supplementary material for: Establishment and Application of Mismatch Amplification Mutation Assay-PCR for Rapid Detection and Differentiation of Duck Hepatitis A Virus-1 Attenuated Vaccine and Wild Strains
Source: Animals (Basel). 2024 Sep 21;14(18):2733. doi: 10.3390/ani14182733 (PMC11428521; doi:10.3390/ani14182733)
Supplement: Supplementary file 1 [file animals-14-02733-s001.zip › animals-3182364-supplementary.pdf]

Supplement Table S1. Comparison of VP1 gene nucleotide-specific mutation sites among ATCC strain, Korean wild strains, Korean vaccine strain and clinical strains

Strain name	Strain type	Nucleotide position			Accession numbers
		2111	2276	2780	
ATCC: VR-1313	ATCC strain (wild)	T	A	A	DQ219396
DHAV-HS	Korean wild strain	T	A	A	DQ812094
DHAV-HSS	Korean wild strain	T	A	A	DQ812092
HSB-P100	Korean vaccine strain	G	G	G	PP592357
D14-MR-056	Clinical strain	G	G	G	PP949248
D15-MR-038	Clinical strain	G	G	G	PP949250
D18-CFR-001	Clinical strain	G	G	G	PP949249
D18-ETC-001	Clinical strain	G	G	G	PP949251

Supplement Table S2. Summary of characteristics of 4 clinically positive samples

Farm	Sample type	Age (days)	Vaccination status	Anatomical symptoms	Other pathogen diagnosic results	Diagnostic result
D14-MR-056	Dead duck	8	Vaccinated at 1 day of age	Hepatic ischemia; Bursal hemorrhage; Splenomegaly; Pancreatic hemorrhage; Uric acid deposition	DEV, DuCV, RA, *Salmonella*, APEC negative	DHAV-1 positive (vaccine strain)
D15-MR-038	Dead duck	9	Unknown	Hepatic punctate hemorrhage; Renal hemorrhage	RA, *Salmonella*, APEC negative	DHAV-1 positive (vaccine strain)
D18-CFR-001	Dead duck	9	Vaccinated at 1 day of age	Hepatic punctate hemorrhage; Hepatic patchy hemorrhage; Renal hemorrhage	*Salmonella* negative	DHAV-1 positive (vaccine strain)
D18-ETC-001[a]	Dead duck	13	Unvaccinated	Hepatic ischemia; Renal ischemia, edema; Uric acid deposition	DuCV, RA, APEC negative; *Salmonella* positive	DHAV-1 positive (vaccine strain); *S.* Typhimurium positive

Abbreviations: DHAV-1, Duck hepatitis A virus 1; DEV, Duck enteritis virus; DuCV, Duck circovirus; RA, *Riemerella anatipstife*r; APEC, Avian Pathogenic *Escherichia coli*; *S.* Typhimurium, *Salmonella* Typhimurium

[a] The ducks from farm of D18-ETC-001 was not vaccinated, vaccination records indicate that a previous batch of ducks raised on the farm had been vaccinated with a live attenuated DHAV-1 vaccine (approximately 60 days ago).
